# Analytical performance of the ScreenFire HPV RS Zebra BioDome assay on four different qPCR platforms

**DOI:** 10.1186/s13027-025-00651-5

**Published:** 2025-04-30

**Authors:** Jun Wang, Godwin Imade, Alani S. Akanmu, Jonah Musa, Rose Anorlu, Yinan Zheng, Brian Joyce, Isaac Adewole, Imran O. Morhason-Bello, Jerome Belinson, Mamoudou Maiga, Demirkan B. Gursel, Atiene S. Sagay, Folasade T. Ogunsola, Robert L. Murphy, Lifang Hou

**Affiliations:** 1 Department of Preventive Medicine, Division of Cancer Epidemiology and Prevention, Feinberg School of Medicine, Northwestern University, Chicago, USA; 2 Center for Global Oncology, Robert J. Havey Institute for Global Health, Feinberg School of Medicine, Northwestern University, Chicago, USA; 3 Department of Obstetrics and Gynecology, College of Health Sciences, University of Jos, Jos, Nigeria; 4 Department of Hematology and Blood Transfusion, College of Medicine, Lagos University Teaching Hospital, University of Lagos, Lagos, Nigeria; 5 Department of Obstetrics and Gynecology, College of Medicine, University of Lagos, Lagos, Nigeria; 6 HPV Research Consortium, College of Medicine, University of Ibadan, Ibadan, Nigeria; 7 Department of Obstetrics and Gynaecology, and HPV Research Consortium, College of Medicine, University of Ibadan, Ibadan, Nigeria; 8 Women’s Health Institute, Cleveland Clinic, and Preventive Oncology International, Inc., Cleveland, USA; 9 Department of Pathology, Feinberg School of Medicine, Northwestern University, Chicago, USA; 10 Department of Medical Microbiology, College of Medicine, University of Lagos, Lagos, Nigeria; 11 Division of Infectious Diseases, Department of Medicine, Feinberg School of Medicine, Northwestern University, Chicago, USA; 12 Robert J. Havey MD Institute for Global Health, Northwestern University, Chicago, USA

**Keywords:** High-risk human papillomavirus (hr-HPV), ScreenFire HPV Zebra biodome assay, iAMP-PS96 personal station qPCR system, Powergene9600 plus Real-Time PCR system

## Abstract

**Objectives:**

Cervical cancer is one of the most frequently diagnosed cancers and a leading cause of cancer-related deaths in women in low- and middle-income countries (LMICs), accounting for nearly 85% of the global cervical cancer burden. High-risk human papillomavirus (hrHPV) infection is the main cause of cervical cancer. Easy-to-use, rapid, scalable, high-throughput, and cost-effective HPV tests are urgently needed for low-resource settings. Atila Biosystems’ clinically validated ScreenFire HPV Risk Stratification (RS) assay identifies 13 hrHPV in 4 groups based on their oncogenic risk (i.e., HPV16, HPV18/45, HPV31/33/35/52/58, and HPV51/59/39/56/68). While the current standard format is subject to laboratory contamination Atila has developed an innovative, contamination-preventive Zebra BioDome format. Recently we published the analytical performance of ScreenFire RS Zebra BioDome on the BioRad CFX-96 real-time PCR instrument. This current study evaluated its analytical performance on three additional qPCR platforms: Atila Portable iAMP-PS96, Atila Powergene9600 Plus, and Thermo Fisher Quantstudio-7.

**Methods:**

We tested 173 DNA samples from Nigerian women with cervical cancer. These samples were tested simultaneously using the ScreenFire HPV Zebra BioDome assay (M5FHPV-96) on four different real-time PCR machines (Atila portable iAMP-PS96, Atila Powergene9600 Plus, Thermo Fisher QuantStudio-7, and BioRad CFX-96). We used overall agreement rate and unweighted kappa values to compare different platforms.

**Results:**

The overall agreement for detection of hrHPV using Atila portable iAMP-PS96 was 96.5% with kappa value 0.95 (95% confidence interval: 0.91–0.99) compared to Thermo Fisher QuantStudio-7, and 97.1% with kappa value 0.96 (95% confidence interval: 0.92–0.99) compared to BioRad CFX-96. For genotype HPV16 and risk stratification (RS) genotype groups (HPV18/45, HPV31/33/35/52/58, and HPV51/59/39/56/68) agreement rates were all > 98.3%. For Atila Powergene9600 Plus the overall agreement was 98.8% with a kappa value of 0.98 (95% confidence interval: 0.96–1.0) compared to Thermo Fisher QuantStudio-7, and 96.5% with a kappa value of 0.96 (95% confidence interval: 0.94–0.99) compared to BioRad CFX-96. The agreements for the HPV16 and RS genotype groups (HPV18/45, HPV31/33/35/52/58, and HPV39/51/56/59/68) were at least 98.3%.

**Conclusion:**

The novel ScreenFire HPV Zebra BioDome format produced highly concordant hrHPV positivity and RS genotype results on all four qPCR platforms. The data suggests that this innovative technology has the potential to improve HPV testing uptake in low-resource settings without further investment in purchasing new equipment.

## Introduction

Globally, cervical cancer is one of the most frequently diagnosed cancers and a leading cause of cancer-related deaths in women in low- and middle-income countries (LMICs) which accounts for nearly 85% of the global cervical cancer burden [1, 2]. Cervical cancer is driven by the persistence of high-risk human papillomavirus (hrHPV) infection, the most significant risk factor for the development of cervical cancer [3, 4]. In 2022, the World Health Organization (WHO) released new guidance shifting primary cervical cancer screening recommendations to HPV DNA testing in all settings, and away from unaided visual inspection using acetic acid (VIA) [5]. Current HPV genotyping assays used in LMICs have a variety of limitations: they are time-consuming, labour-intensive, costly, lacking high-throughput capabilities, and at risk of laboratory contamination [6–8]. This latter risk is particularly common in PCR-based assays, and hinders the adoption of these assays for large-scale hrHPV testing in low-resource areas without a standard (i.e., costly) laboratory setup and available expert personnel.

Among the existing assays, Atila Biosystems’ clinically validated ScreenFire HPV RS assay (M5FHPV-100) has been specifically designed for use in LMIC cervical cancer screening programs [9–11]. It provides hierarchal risk stratification (RS) genotyping information by identifying 13 hrHPV genotypes in 4 groups based on their oncogenic risk (i.e., HPV16, HPV18/45, HPV31/33/35/52/58, and HPV51/59/39/56/68). The ScreenFire HPV RS assay uses isothermal amplification and reports the four hrHPV channels using fluorescent detection, with channel sensitivity designed according to hierarchical cancer risk. It does so at a low cost per test, with high throughput and a reported overall sensitivity of 94.7%.^9,10^ The ScreenFire HPV RS assay offers high capacity in standard 96-well plates with less than one hour of processing time. In addition, it is particularly suited for primary cervical cancer screening since collected specimens do not require DNA extraction and purification [11]. However, the current format of the ScreenFire HPV RS assay still relies on manual preparation of the master mix of reagents [12–15], which requires a standard molecular laboratory setup and well-trained laboratory personnel to minimize possible laboratory contamination.

To address these challenges, Atila Biosystems has developed a new format for the ScreenFire HPV RS assay: the ScreenFire HPV RS assay Zebra BioDome (M5FHPV-96). This novel format features pre-loaded reagents in either 8-well PCR strips or 96-well PCR plates, covered by a temperature-sensitive hydrophobic gel matrix. The hydrogel matrix in the reagent tube is highly stable and semi-solidified during transportation and storage before full implementation. This protects the reagents from leakage or spillage and contaminating the laboratory. The matrix liquefies and moves to the top of the liquid to seal the reaction wells during isothermal heating and re-solidifies after amplification and before disposal. Thus, in addition to the common features shared with the ScreenFire Standard format (e.g. easy to use, high-throughput, cost-appropriate in low-resource settings, and no requirements for DNA extraction) the contamination-prevention feature along with the lack of required reagent preparation makes this Zebra BioDome format highly desirable. It only requires the single step of adding the patient’s sample before the remaining automated process, thus making it particularly suitable for large-scale cervical cancer screening via primary HPV screening.

In our recently published data, we reported that the Zebra BioDome format generated highly concordant results compared to the standard ScreenFire HPV RS assay when the BioRad CFX-96 PCR instrument was used [12]. The overall agreement for detection of hrHPV was 96.0%. The agreement rates between hrHPV genotype 16 and risk stratification genotype group (HPV18/45, HPV31/33/35/52/58, and HPV51/59/39/56/68) were all > 97.5%. The U.S. National Cancer Institute has also reported that the Zebra BioDome performed similarly to the standard version of the ScreenFire HPV assay using Atila Powergene Instrument [7]. Specifically, Zebra BioDome showed agreement with the standard version in the channel-specific analysis with positive percent agreement between 88.4% and 100% and negative percent agreement between 97.8% and 100%, as well as in hierarchical analysis with overall agreement 97.2%. This validation strengthened the case for wider adoption of the ScreenFire BioDome assay. However, many laboratories in LMICs may already have other PCR instruments (e.g., the common Thermo Fisher QuantStudio-7 and BioRad CFX-96). For this study we evaluated the analytical performance of the ScreenFire RS hrHPV assay on four qPCR platforms: Atila iAMP-PS96, Atila Powergene9600 Plus, Thermo Fisher Quantstudio-7, and BioRad CFX-96.

## Materials and methods

### Study samples

This study is built upon our NCI-funded U54 consortium to study Epigenomic Biomarkers of HIV-Associated Cancers in Nigeria (U54CA221205). In total, the 173 cervical tissue samples used in this study were collected between 2018 and 2022 in Nigerian women diagnosed with cervical cancer [16]. The age range was 26 to 80 years. The use of these samples was covered under IRB approval at Northwestern University, Jos University, and Lagos University. The samples were obtained at the Jos University Teaching Hospital (JUTH) and Lagos University Teaching Hospital (LUTH) in Nigeria. The further HPV testing was allowed in the informed consent and the results available to all women who underwent testing. DNA was extracted from the cervical biopsies using the Qiagen QIAamp DNA Mini Kit and quantified using Qubit 4.0 fluorometer. DNA samples were stored at − 80 C until shipment. All DNA samples were de-identified when shipped in dry ice to Northwestern University and stored at − 80 C.

### Detection and RS genotyping of HrHPV

A total of 100ng purified DNA was prepared in 100µL of 1X lysis buffer and processed following procedures for hrHPV RS genotyping using the ScreenFire RS Zebra BioDome HPV test kits purchased from Atila BioSystems, Inc (Atila, Sunnyvale, CA). The prepared 10 µl DNA samples were added into the prepacked Zebra BioDome reaction tubes. The capped reaction tubes were spun for 10 s to bring all the liquid down to the bottom. The strips were then loaded into four different real-time PCR machines (Atila iAMP-PS96, Atila Powergene9600 Plus, Thermo Fisher QuantStudio-7 or BioRad CFX-96) and the assays were carried out on the isothermal program mode run at 1 min per cycle at 60℃ for 60 cycles. Fluorescence was obtained from CY5 (for HPV16), ROX (for HPV18/45), CY5.5 (for HPV31/33/35/52/58), FAM (for HPV39/51/56/59/68) and HEX (for human beta globin gene as internal control). A sample was considered positive for the corresponding HPV genotype if the signal was detected within 60 min in the channel, regardless of the signal in the HEX channel. If no signal was detected for any of the four HPV channels within 60 min, then a signal was required in the HEX channel for the batch run to be called a valid negative.

### Statistical analysis

The performance of the ScreenFire HPV Zebra BioDome assay on Atila iAMP-PS96 and Atila Powergene9600 Plus was evaluated based on the consistency with Thermo Fisher QuantStudio-7 and BioRad CFX-96. The data was assessed according to positive, negative, and overall agreement; as well as the unweighted kappa values for the 13 HPV genotypes (HPV16, 18, 31, 33, 35, 39, 45, 51, 52, 56, 58, 59 and 68) in the four detection groups. The groups (channels) were analysed hierarchically based on cervical cancer risk: HPV16 positive, else positive for HPV18/45, else positive for HPV 31/33/35/52/58, else positive for HPV51/59/39/56/68, or else negative.

## Results

On the pairwise hierarchical analysis, Atila iAMP-PS96 showed an overall agreement of 96.5% (167/173) with a kappa value of 0.95 (95% confidence interval: 0.91–0.99) compared to the results from Thermo Fisher QuantStudio-7 (Table 1A). Similarly, when compared to the results from BioRad CFX-96, the overall agreement was 97.1% (168/173) with a kappa value of 0.96 (95% confidence interval: 0.92–0.99, Table 1B). The detailed agreement for HPV RS genotyping is shown in Table 2A. The respective agreement rates for the genotypes of HPV16, HPV18/45, HPV31/33/35/52/58, and HPV39/51/56/59/68 were 99.4%, 98.3%, 99.4%, and 98.8%; the corresponding kappa values were 0.98 (95% confidence interval 0.95-1.0), 0.94 (0.86-1.0), 0.97 (0.91-1.0), and 0.90 (0.77-1.0) respectively. The agreement rates for RS genotypes when comparing iAMP-PS96 vs. CFX-96 were at least 98.8% (Table 2B). Thus, the Atila iAMP-PS96 demonstrated highly consistent results when compared to well-established qPCR technologies, such as Thermo Fisher QuantStudio-7 and BioRad CFX-96, using the ScreenFire RS Zebra BioDome HPV assays.

The pairwise hierarchical analysis between the results on Atila Powergene9600 Plus and Thermo Fisher QuantStudio-7 also showed high consistency with an overall agreement of 98.8% (171/173) with a kappa value of 0.98 (95% confidence interval: 0.96–1.0, Table 3A). When the results on Atila Powergene9600 Plus were compared to those from BioRad CFX-96, the overall agreement of 96.5% (167/173) with a kappa value of 0.96 (95% confidence interval: 0.94–0.99, Table 3B). As shown in Table 4A, the agreement rate for HPV16 genotype was 98.8% with a kappa value of 0.97 (95% confidence interval 0.92-1.0) when comparing Atila Powergene9600 Plus and Thermo Fisher QuantStudio-7. The agreement for RS genotypes of HPV18/45, HPV31/33/35/52/58, and HPV39/51/56/59/68 were all 100%. The agreement rates for respective RS genotypes were at least 98.3% when comparing Atila Powergene9600 Plus and BioRad CFX-96 (Table 4B). Thus, Atila Powergene9600 Plus also demonstrated highly consistent results when compared to Thermo Fisher QuantStudio-7 and BioRad CFX-96 using the ScreenFire RS Zebra BioDome HPV assay.

## Discussion

Understanding the analytical performance of the ScreenFire HPV RS Zebra BioDome assay on commonly used qPCR instruments, not only from Atila but also from other manufacturers, will provide important information for the future use of this innovative HPV DNA test for cervical cancer screening. As the widely used clinically validated HPV assays on the market are mostly closed systems that require dedicated platforms, such as GeneXpert (cartridge-based), Roche Cobas, BD Onclarity and Seegene Allplex, so far none of the clinical validation studies has been done on the lab existing qPCR equipment. In contrast, the Atila ScreenFire HPV assay is an open-platform solution, allowing for broader compatibility. It has been evaluated and compared to other qPCR-based HPV genotyping assays on specific devices separately [9–15]. To our knowledge this is the first study to evaluate Zebra BioDome format across multiple existing platforms simultaneously. This study verified ScreenFire Zebra BioDome HPV assays are compatible with widely used platforms existed in the lab.

The ScreenFire Zebra BioDome HPV assays not only offers the compatibility capability but also helps to address many other hurdles or limitations for implementing cervical cancer screening in low resource constrained settings, such as the needs for trained laboratory personnel and a dedicated laboratory setting to prevent contamination. The ScreenFire HPV RS Zebra BioDome format greatly reduced the number of processing steps, which now only involves adding lysed samples to pre-loaded Zebra BioDome reaction tube strips. The resulting process is fast, easy-to-use and high throughput, besides virtually eliminating the risk of laboratory contamination. In addition, the Zebra BioDome assay is also resistant to environmental fluctuations, as its performance remains unaffected by environmental temperature variations and other external conditions.

This study provides the first scientific data to show that the Zebra BioDome assays can be used in several commonly used qPCR platforms for HPV DNA testing. This cross-platform applicability has high public health significance in order to remove financial burden of capital investment to purchase new equipment for implementation cervical cancer screening. Our study in Mali and Nigeria has demonstrated that a robust and sustained implementation of a community-based ScreenFire Zebra BioDome HPV detection system using self-collected samples and operated by minimum-laboratory trained persons is feasible in LMIC settings.

Besides the unique features that have been described above, the ScreenFire Zebra BioDome HPV detection system is one of the most affordable HPV tests on the market compared to other commercially available HPV screening options. The ScreenFire HPV RS assay with Zebra BioDome technology is currently priced at $5.95 per test and the iAMP-PS96 device costs $13,500, about one-quarter the cost of the most common existing platforms. The low cost per test combined with the portable and battery-operated nature of the iAMP-PS96 platform makes it a highly cost-effective solution for large-scale cervical cancer screening programs, particularly in low-resource settings. This study shows the value of ScreenFire HPV technology to make the WHO’s goal to screen 70% women in the world closer to a reality.

**Table 1:** Pairwise comparison between the HPV detection results of Atila iAMP-PS96 and (A) Thermo Fisher QuantStudio-7 or (B) BioRad CFX-96 using Screenfire HPV Zebra BioDome assay categorized hierarchically according to HPV RS genotypes

(A) iAMP-PS96 vs. QuantStudio-7						
iAMP-PS96	QuantStudio-7					
	HPV16	HPV18/45	HPV31/33 /35/52/58	HPV39/51 /56/59/68	Negative	Total
**HPV16**	**36**	0	0	0	1	**37**
Row %	**97.3**	0	0	0	2.7	**100**
Column %	**100**	0	0	0	1.2	**21.4**
**HPV18/45**	0	**26**	1	0	1	**28**
Row %	0	**92.8**	3.6	0	3.6	**100**
Column %	0	**96.3**	5	0	1.2	**16.2**
**HPV31/33/35 /52/58**	0	0	**19**	0	0	**19**
Row %	0	0	**100**	0	0	**100**
Column %	0	0	**95**	0	0	**11**
**HPV39/51/56 /59/68**	0	0	0	**10**	2	**12**
Row %	0	0	0	**83.3**	16.7	**100**
Column %	0	0	0	**100**	2.6	**6.9**
**Negative**	0	1	0	0	**76**	**77**
Row %	0	1.3	0	0	**98.7**	**100**
Column %	0	3.7	0	0	**95**	**44.5**
**Total**	**36**	**27**	**20**	**10**	**80**	**173**
Row %	20.8	15.6	11.6	5.8	46.2	**100**
Column %	100	100	100	100	100	**100**
**Overall agreement rate = 96.5% (167/173); Unweighted kappa (95% CI) = 0.95 (0.91**,** 0.99)**						

(B) iAMP-PS96 vs. CFX-96						
iAMP-PS96	CFX-96					
	HPV16	HPV18/45	HPV31/33 /35/52/58	HPV39/51 /56/59/68	Negative	Total
**HPV16**	**36**	0	0	0	1	**37**
Row %	**97.3**	0	0	0	2.7	**100**
Column %	**97.3**	0	0	0	1.3	**21.4**
**HPV18/45**	0	**27**	1	0	0	**28**
Row %	0	**96.4**	3.6	0	0	**100**
Column %	0	**96.4**	5	0	0	**16.2**
**HPV31/33/35 /52/58**	0	0	**19**	0	0	**19**
Row %	0	0	**100**	0	0	**100**
Column %	0	0	**95**	0	0	**11**
**HPV39/51/56 /59/68**	0	0	0	**11**	1	**12**
Row %	0	0	0	**91.7**	8.3	**100**
Column %	0	0	0	**100**	1.3	**6.9**
**Negative**	1	1	0	0	**75**	**77**
Row %	1.3	1.3	0	0	**97.4**	**100**
Column %	2.7	3.6	0	0	**97.4**	**44.5**
**Total**	**37**	**28**	**20**	**11**	**77**	**173**
Row %	21.4	16.2	11.6	6.4	44.5	**100**
Column %	100	100	100	100	100	**100**
**Overall agreement rate = 97.1% (168/173); Unweighted kappa (95% CI) = 0.96 (0.92**,** 0.99)**						

**Table 2:** Agreement between Atila iAMP-PS96 and (A) Thermo Fisher QuantStudio-7 or (B) BioRad CFX-96 using Screenfire HPV Zebra BioDome assay for HPV RS genotyping

(A) iAMP-PS96 vs. QuantStudio-7								
	+/+ *n*(%)	-/+ *n*(%)	+/- *n*(%)	-/- *n*(%)	Positive agreement % (95% CI)	Negative agreement % (95% CI)	Overall agreement % (95% CI)	Unweighted kappa (95% CI)
HPV16	36 (20.8)	0 (0)	1 (0.6)	136 (78.6)	100 (90.3–100)	99.3 (96–100)	99.4 (96.8–100)	0.98 (0.95-1)
HPV18/45	26 (15)	1 (0.6)	2 (1.2)	144 (83.2)	96.3 (81-99.9)	98.6 (95.1–99.8)	98.3 (95-99.6)	0.94 (0.86-1)
HPV31/33 /35/52/58	19 (11)	1 (0.6)	0 (0)	153 (88.4)	95 (75.1–99.9)	100 (97.6–100)	99.4 (96.8–100)	0.97 (0.91-1)
HPV39/51 /56/59/68	10 (5.8)	0 (0)	2 (1.2)	161 (93.1)	100 (69.2–100)	98.8 (95.6–99.9)	98.8 (95.9–99.9)	0.90 (0.77-1)

(B) iAMP-PS96 vs. CFX-96								
	+/+ *n*(%)	-/+ *n*(%)	+/- *n*(%)	-/- *n*(%)	Positive agreement % (95% CI)	Negative agreement % (95% CI)	Overall agreement % (95% CI)	Unweighted kappa (95% CI)
HPV16	36 (20.8)	1 (0.6)	1 (0.6)	135 (78)	97.3 (85.8–99.9)	99.3 (96–100)	98.8 (95.9–99.9)	0.97 (0.92-1)
HPV18/45	27 (15.6)	1 (0.6)	1 (0.6)	144 (83.2)	96.4 (81.7–99.9)	99.3 (96.2–100)	98.8 (95.9–99.9)	0.96 (0.9-1)
HPV31/33 /35/52/58	19 (11)	0 (0)	1 (0.6)	153 (88.4)	100 (82.4–100)	99.4 (96.4–100)	99.4 (96.8–100)	0.97 (0.91-1)
HPV39/51 /56/59/68	11 (6.4)	1 (0.6)	0 (0)	161 (93.1)	91.7 (61.5–99.8)	100 (97.7–100)	99.4 (96.8–100)	0.95 (0.86-1)

**Table 3:** Pairwise comparison between the HPV detection results of Atila PowerGene9600 plus and (A) Thermo Fisher QuantStudio-7 or (B) BioRad CFX-96 using Screenfire HPV Zebra BioDome assay categorized hierarchically according to HPV RS genotypes

(A) PowerGene9600 plus vs. QuantStudio-7						
PowerGene9600 Plus	QuantStudio-7					
	HPV16	HPV18/45	HPV31/33 /35/52/58	HPV39/51 /56/59/68	Negative	Total
**HPV16**	**36**	0	0	0	2	**38**
Row %	**94.7**	0	0	0	5.3	**100**
Column %	**100**	0	0	0	2.5	**22**
**HPV18/45**	0	**27**	0	0	0	**27**
Row %	0	**100**	0	0	0	**100**
Column %	0	**100**	0	0	0	**15.6**
**HPV31/33/35** **/52/58**	0	0	**20**	0	0	**20**
Row %	0	0	**100**	0	0	**100**
Column %	0	0	**100**	0	0	**11.6**
**HPV39/51/56** **/59/68**	0	0	0	**10**	0	**10**
Row %	0	0	0	**100**	0	**100**
Column %	0	0	0	**100**	0	**5.8**
**Negative**	0	0	0	0	**78**	**78**
Row %	0	0	0	0	**100**	**100**
Column %	0	0	0	0	**97.5**	**45.1**
**Total**	**36**	**27**	**20**	**10**	**80**	**173**
Row %	20.8	15.6	11.6	5.8	46.2	**100**
Column %	100	100	100	100	100	**100**
**Overall agreement rate = 98.8% (171/173); Unweighted kappa (95% CI) = 0.98 (0.96**,** 1)**						

(B) PowerGene9600 Plus vs. CFX-96						
PowerGene9600 Plus	CFX-96					
	HPV16	HPV18/45	HPV31/33 /35/52/58	HPV39/51 /56/59/68	Negative	Total
**HPV16**	**36**	0	0	0	1	**37**
Row %	**97.3**	0	0	0	2.7	**100**
Column %	**100**	0	0	0	1.2	**21.4**
**HPV18/45**	0	**26**	1	0	1	**28**
Row %	0	**92.8**	3.6	0	3.6	**100**
Column %	0	**96.3**	5	0	1.2	**16.2**
**HPV31/33/35** **/52/58**	0	0	**19**	0	0	**19**
Row %	0	0	**100**	0	0	**100**
Column %	0	0	**95**	0	0	**11**
**HPV39/51/56** **/59/68**	0	0	0	**10**	2	**12**
Row %	0	0	0	**83.3**	16.7	**100**
Column %	0	0	0	**100**	2.6	**6.9**
**Negative**	0	1	0	0	**76**	**77**
Row %	0	1.3	0	0	**98.7**	**100**
Column %	0	3.7	0	0	**95**	**44.5**
**Total**	**36**	**27**	**20**	**10**	**80**	**173**
Row %	20.8	15.6	11.6	5.8	46.2	**100**
Column %	100	100	100	100	100	**100**
**Overall agreement rate = 96.5% (167/173); Unweighted kappa (95% CI) = 0.96 (0.94**,** 0.99)**						

**Table 4:** Agreement between Atila PowerGene9600 plus and (A) Thermo Fisher QuantStudio-7 or (B) BioRad CFX-96 using Screenfire HPV Zebra BioDome assay for HPV RS genotyping

(A) PowerGene9600 plus vs. QuantStudio-7								
	+/+ *n*(%)	-/+ *n*(%)	+/- *n*(%)	-/- *n*(%)	Positive agreement % (95% CI)	Negative agreement % (95% CI)	Overall agreement % (95% CI)	Unweighted kappa (95% CI)
HPV16	36 (20.8)	0 (0)	2 (1.2)	135 (78)	100 (90.3–100)	98.5 (94.8–99.8)	98.8 (95.9–99.9)	0.97 (0.92-1)
HPV18/45	27 (15.6)	0 (0)	0 (0)	146 (84.4)	100 (87.2–100)	100 (97.5–100)	100 (97.9–100)	1 (1–1)
HPV31/33 /35/52/58	20 (11.6)	0 (0)	0 (0)	153 (88.4)	100 (83.2–100)	100 (97.6–100)	100 (97.9–100)	1 (1–1)
HPV39/51 /56/59/68	10 (5.8)	0 (0)	0 (0)	163 (94.2)	100 (69.2–100)	100 (97.8–100)	100 (97.9–100)	1 (1–1)

(B) PowerGene9600 Plus vs. CFX-96								
	+/+ *n*(%)	-/+ *n*(%)	+/- *n*(%)	-/- *n*(%)	Positive agreement % (95% CI)	Negative agreement % (95% CI)	Overall agreement % (95% CI)	Unweighted kappa (95% CI)
HPV16	36 (20.8)	2 (1.2)	1 (0.6)	134 (77.5)	94.7 (82.3–99.4)	99.3 (95.9–100)	98.3 (95-99.6)	0.95 (0.89-1)
HPV18/45	26 (15)	1 (0.6)	2 (1.2)	144 (83.2)	96.3 (81-99.9)	98.6 (95.1–99.8)	98.3 (95-99.6)	0.94 (0.86-1)
HPV31/33 /35/52/58	20 (11.6)	0 (0)	0 (0)	153 (88.4)	100 (83.2–100)	100 (97.6–100)	100 (97.9–100)	1 (1–1)
HPV39/51 /56/59/68	10 (5.8)	0 (0)	1 (0.6)	162 (93.6)	100 (69.2–100)	99.4 (96.6–100)	99.4 (96.8–100)	0.95 (0.85-1)

## Data Availability

No datasets were generated or analysed during the current study.
